# Meatal stenosis and lichen sclerosus in children: is it a real risk? A single-centre retrospective observational study

**DOI:** 10.1308/rcsann.2024.0096

**Published:** 2025-02-28

**Authors:** R Angotti, F Nascimben, M Messina, A Roggero, N Calomino, V Bagnara, F Raffaelli, F Ferrara, A Benigna, F Molinaro

**Affiliations:** ^1^ Division of Pediatric Surgery, Department of Medical, Surgical and Neurosurgical Sciences, Policlinico Le Scotte, Siena, Italy; ^2^ Policlinico G.B.Morgagni, Italy

**Keywords:** Lichen Sclerosus, Meatal stenosis, Children, Circumcision, Phimosis

## Abstract

**Background:**

Early diagnosis, early treatment and long-term follow-up in paediatric patients with Lichen Sclerosus (LS) are mandatory to avoid complications such as urethral meatal stenosis.

**Methods:**

All patients older than five years who underwent circumcision from January 2015 to December 2021 at our centre with positive histology for LS were included. Demographic, preoperative, surgical and postoperative data were analysed. Patients were physically evaluated, and they were asked to fill in two quality of life questionnaires and to perform an uroflowmetry. They were stratified into clusters according to physical and histological examination. Urethral dilatations were investigated to assess the correlation between circumcision and incidence of LS-linked complications.

**Results:**

Among 99 patients included in the study, 95 were finally evaluated. Median age at diagnosis was seven years (range, five to ten years). Median age at surgery was 10.8 years (6–17). Urethral meatus was grade 0 in 47% of cases, grade 1 in 41% and grade 2 in 12%. A total of 19% of circumcised patients with LS had pathological uroflowmetry: the number of patients with pathological uroflowmetry increased as the grade of meatal stenosis increased (13% grade 0, 15% grade 1 and 33% grade 2). Four (4.7%) patients with diagnosis of meatal stenosis underwent meatal dilatations.

**Conclusions:**

By assessing histology of LS it is possible to determine who will develop LS-linked complications such as meatal stenosis. Patients with LS must be followed-up closely and should be treated with corticosteroids for at least for one month to improve their postoperative outcomes.

## Introduction

Lichen Sclerosus (LS), also known as Balanitis Xerotica Obliterans, is a chronic inflammatory pathology, the aetiology of which is not yet completely understood.^1^ LS may develop at any age, with no preference for sex or race.^2,3^ The precise incidence is unknown and difficult to estimate due to the heterogeneity of the disease; however, it is estimated to affect approximately 0.003% of the world’s population^1^ and about 0.1–0.7% of prepubertal children.^3^ Although it may occur at any cutaneous site, the anogenital area is privileged.^1^ In paediatric male patients, the main manifestation of LS is phimosis. While asymptomatic in 24% of cases, the remaining 76% present with nonspecific signs such as whitish or red areas on the glans penis, a thickened foreskin or coronal sulcus.^2^ LS-associated phimosis is one of the most frequent causes of urethral stricture,^4^ with early (postoperative bleeding, wound infections), and long-term consequences (dysuria, pollakiuria, meatal stenosis, redness on the glans penis, thin urinary flow, prolonged urinary flow and urinary hesitance). Here, we report our experience and compare our data with the literature.

The aim of this study was to investigate the link between LS and meatal stenosis in patients in the paediatric age group who underwent circumcision. After demonstrating there is a link, we would like to show that an early diagnosis, early treatment and long-term follow-up for paediatric patients with LS are mandatory to avoid severe complications such as urethral meatal stenosis.

## Methods

We carried out a single-centre retrospective observational study, including all paediatric patients older than six years of age who presented with pathological phimosis surgically treated by circumcision and who had a histopathological diagnosis of LS, at the Department of Paediatric Surgery at the University Hospital of Siena, between January 2015 and December 2021. Patients younger than six years old, patients who underwent ritual circumcision, patients without a histopathological diagnosis of LS and patients without a minimum follow-up of six months were excluded from the study. Data were extracted from the hospital medical record database. For all patients, we documented the age at diagnosis, presenting symptoms, topical treatment before surgery, age at surgery, intraoperative and postoperative complications, postoperative topical treatment, postoperative meatal stenosis and the necessity for urethral dilatation and/or meatoplasty. An ethical committee was set up and approval for the study was obtained. All eligible patients were contacted by telephone. All patients were evaluated in the presence of at least one parent for a clinical interview, physical examination, uroflowmetry and two satisfaction questionnaires. All outpatient visits had a duration of about 20 minutes. The average follow-up time was 51 months (6–84 months).

### Clinical interview

Patients and parents were informed about the study before the interview. Patient medical history was collected and an individual form was completed with personal data, which were then reported anonymously in a database.

### Physical examination

Physical examination focused on the aesthetic appearance of the penis and the features of the urethral meatus. We used a meatal stenosis grading system that included 3 grades^5^: grade 0 (wide open meatus, visible mucosa), grade 1 (minimal mucosa/fibrotic tissue visible) and grade 2 (pinpoint meatus/no mucosa visible/large fibrotic layer). Parents or patients were asked about the urinary stream. The data were collected in a specific form.

### Uroflowmetry

Uroflowmetry parameters considered in the study were: flow pattern, peak urinary flow rate (Qmax), voided volume standardised for age and time to peak and average flow rate (Qmed) standardised for age.^6^ In accordance with Practical Uroflowmetry, Qmax was considered pathological with a value under 10ml/s and borderline with a value between 10 and 15ml/s; voided volume standardised for age was considered pathological if under 65% of the expected bladder capacity in millilitres for age and the Qmed standardised for age was considered pathological with a value under 13ml/s in patients between 6 and 13 years old and under 21ml/s in patients older than 14 years old.^6^

### Satisfaction quality questionnaires

**Figure 1:**
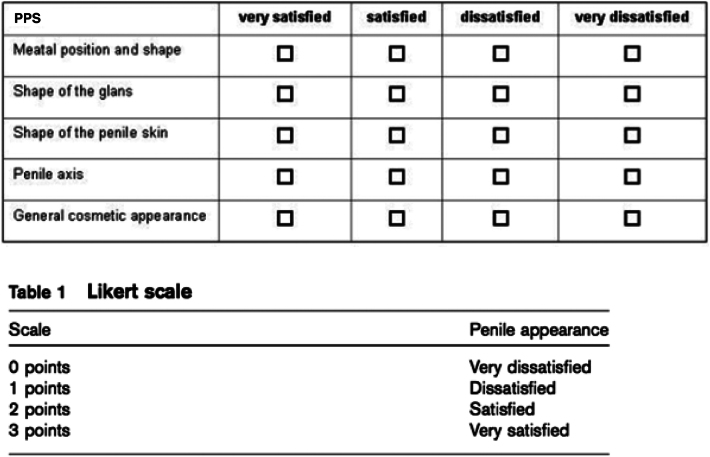
Penile perception score (PPS)^7^

The paediatric penile perception score^7^ was filled out by patients, parents and the surgeon. The score comprises an evaluation of meatal position and appearance, the appearance of the glans penis, the appearance of the shaft of the penis and the general appearance of the penis (Figure 1). According to the paediatric penile perception score, a very satisfactory score was considered between 12 and 10; a satisfactory one between 9 and 6 and a low satisfactory score if under 6. All patients were evaluated by a single doctor who gave them a score based on the paediatric penile perception score.^7^

**Figure 2:**
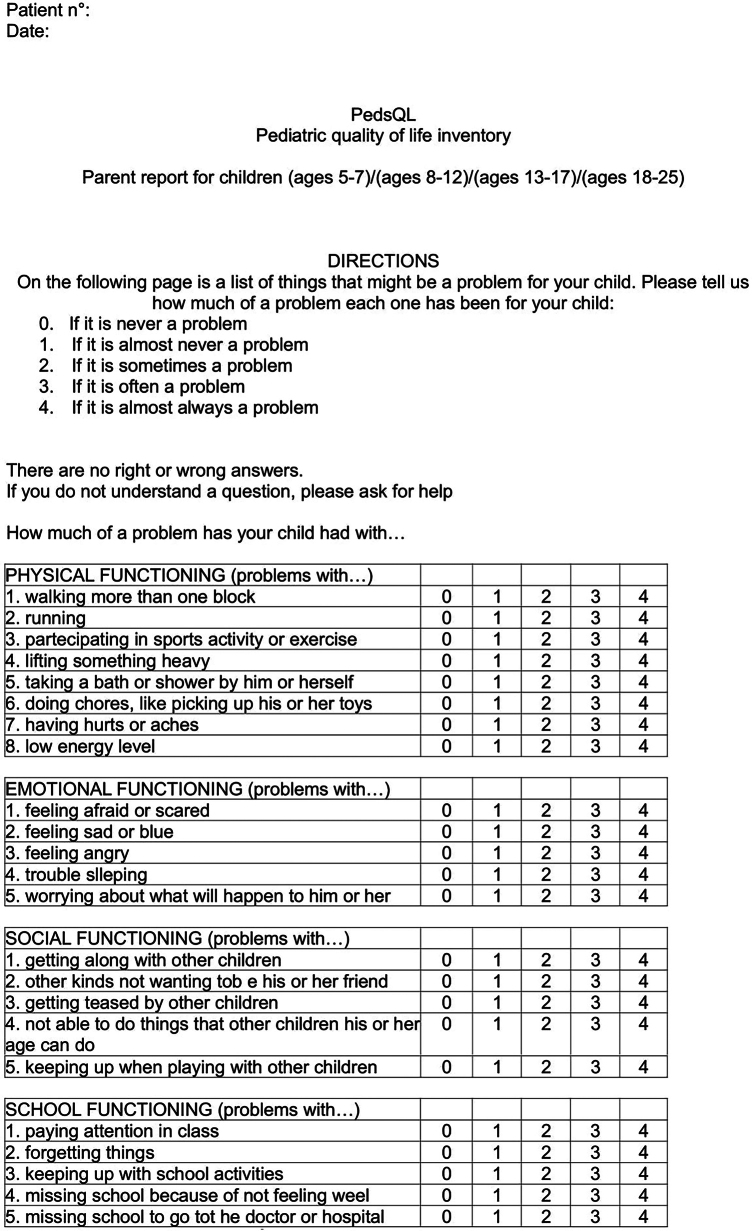

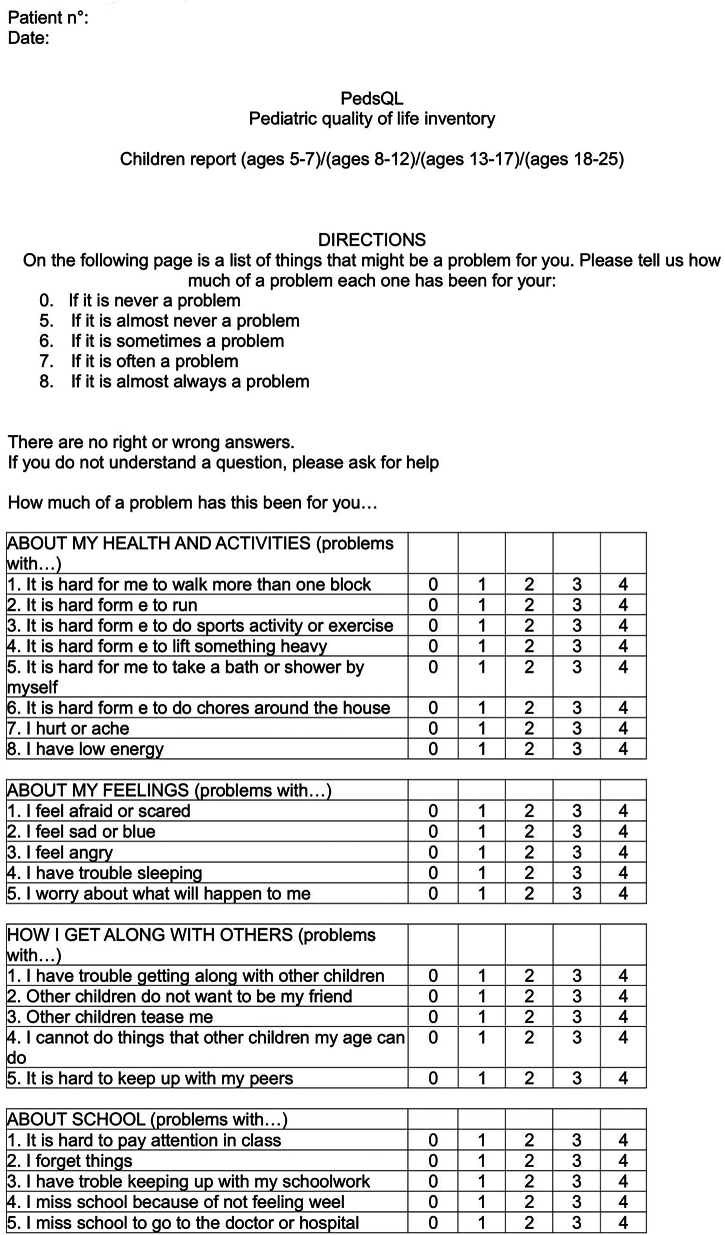
(A) PedsQL parent report.^8^ (B) PedsQL child report.^8^ PedsQL = Pediatric Quality of Life Inventory.

Patients and parents filled out Pediatric Quality of Life Inventory (PedsQL)^8^ (Figure 2A,B). We considered a score over 80% as excellent, a score between 70% and 80% as acceptable, and a score under 70% as poor.^8^

### Statistical analysis

Statistical analysis was performed using GraphPad and R. Continuous variables are presented as mean, median and standard deviation values. According to the normality of the distribution, discrete data are reported as numbers and proportions. Categorical variables are presented as frequencies and percentages. Chi-square or Fisher’s exact test were used to confer categorical data. Independent *t*-test and analysis of variance were used to confer satisfaction scores among two and more than two groups, respectively. A *p*-value <0.05 was considered significant.

## Results

Among 1,273 patients who underwent circumcision between January 2015 and December 2021 at the Paediatric Surgery Department of the University Hospital of Siena, 260 (20%) were eligible for our study. Of these, 42 were unreachable (14 patients had a nonexistent telephone number and 28 patients never answered after at least three attempts). Of the remaining 218 patients, 99 agreed to take part in the study.

All 99 patients were examined; however, only 95 were enrolled because 4 did not perform uroflowmetry. The mean age at diagnosis of phimosis was seven years (range, five to ten years). Symptoms at diagnosis were dysuria, urinary infections with painful urination and/or fever and difficulty in starting flow. A total of 56 patients (59%) were previously treated with one or more cycles of topical corticosteroid therapy before surgery, with an average duration of 6.5 weeks (1–12 weeks). The mean age at the time of surgical intervention was 10.8 years (6–17 years).

With regards to complications, we had one case of postoperative bleeding that required an immediate second look. A total of 13 patients had long-term complications, including one case of dysuria, one case of pollakiuria, one case of redness on the glans penis and one case of hypersensitivity; 4 patients reported a thin urinary flow, 4 patients described a prolonged urinary flow and 1 patient reported urinary hesitance.

A total of 42 (44%) patients received postoperative corticosteroid therapy, with a mean duration of 5.7 weeks (2–24 weeks). Of these patients, 1 had undergone circumcision in 2015, 6 in 2017, 8 in 2018, 6 in 2019, 4 in 2020 and 17 in 2021.

Four patients (4.7%) were diagnosed with meatal stenosis during postoperative follow-up; of these, three underwent serial meatal dilatation under general anaesthesia (mean value of two dilatations). One patient had meatal self-dilatations at home for a duration of one month.

### Physical examination

The urethral meatus of 44 patients (47%) was defined as grade 0, 40 (41%) as grade 1 and 11 (12%) as grade 2.

We investigated the correlation between meatal stenosis grading system and symptoms. Evaluation of the correlation between meatal stenosis grading system and symptoms (Table 1) was partially nullified by meatal dilatation in four patients.

**Table 1:** Correlation between meatal stenosis grading system and symptoms described by patients during clinical examination

Grade	With symptoms	With meatal stenosis (after dilatation)
0	0	1 (2%)
1	3 (7.3%)	2 (4.8%) 1 underwent meatal self-dilatation, 1 underwent 1 dilatation under general anaesthesia
2	3 (25%)	1 (8.3%)

We found a correlation to exist between the meatal stenosis grading system and symptoms described by patients during this clinical control. No patient with grade 0 had symptoms, three patients with grade 1 (7.3%) and three patients (25%) with grade 2 were symptomatic (*p*-value = 0.0019). One patient with grade 0, two patients with grade 1 and one patient with grade 3 underwent meatal dilatation.

### Uroflowmetry

All patients included in the study underwent uroflowmetry assessment.

Concerning the parameters that we analysed:

• Flow pattern: 16 (17%) examinations were pathological, and 4 (4%) were borderline.
• Qmax: 4 (4%) examinations were pathological and 17 (18%) were borderline.
• Both parameters: 19 (20%) examinations were pathological, and 19 (20%) were borderline.

### Satisfaction quality questionnaires

Surgeon evaluation of the paediatric penile perception score is shown in Table 2.

**Table 2:** Paediatric penile perception score

Paediatric penile perception score			
	Surgeon	Patients	Parents
Very satisfactory	50 (52.5%)	38 (40.4%)	34 (36.7%)
Satisfactory	39 (41.5%)	53 (55.5%)	55 (59.1%)
Low satisfactory	6 (6%)	4 (4.2%)	4 (4.2%)

All patients were evaluated by a single surgeon who gave them a score based on the paediatric penile perception score. Of 95 patients, 50 (53%) were assigned a very satisfactory score, 39 (41%) a satisfactory score and 6 (6%) a low satisfactory score.

Patient evaluation of the paediatric penile perception score is shown in Table 2.

All patients filled out the paediatric penile perception score; 38 patients (40%) received a very satisfactory score, 53 (56%) a satisfactory score and 4 (4%) a low satisfactory score.

Parent evaluation of the paediatric penile perception score is shown in Table 2.

All 95 parents filled out the paediatric penile perception score; 36 patients (38%) were assigned a very satisfactory score, 55 (58%) a satisfactory score and 4 (4%) a low satisfactory score.

**Figure 3:**
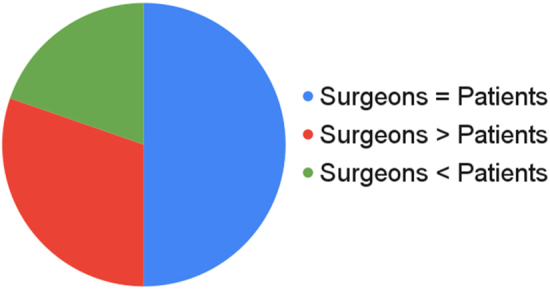
Paediatric penile perception score—patients and surgeon

Correlation between patients and surgeons is shown in Figure 3.

In 48 cases (50%), the score assigned by the surgeon and the patient was in the same range; in 29 cases (30%) the surgeon gave a higher score than the patient; in 18 cases (19%) the surgeon gave a lower score than the patient.

**Figure 4:**
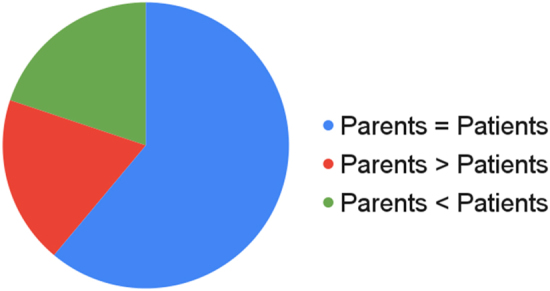
Paediatric penile perception score – patients and parents

Correlation between patients and parents is shown in Figure 4.

In 56 cases (59%), the score assigned by the parent and the patient was in the same range; in 17 cases (18%) the parent's score was higher than the patient's; in 21 cases (19%) the parent's score was lower than the patient's. Patient evaluation of PedsQL is shown in Table 3.

**Table 3:** PedsQL

PedsQL		
	Patients	Parents
Excellent	83 (87.8%)	86 (91.8%)
Acceptable	7 (7.1%)	6 (6.2%)
Poorly acceptable	5 (5.1%)	2 (2%)

PedsQL =  Pediatric Quality of Life Inventory.

All patients filled out the PedsQL; 83 patients (88%) received an excellent score, 7 (7%) an acceptable score and 5 (5%) a poorly acceptable score.

Parent evaluation of PedsQL is shown in Table 3.

All 95 parents filled out the PedsQL; 87 patients (92%) received an excellent score, 6 (6%) an acceptable score and 2 (2%) a poorly acceptable score.

**Figure 5:**
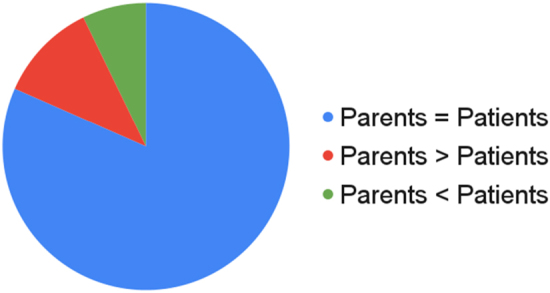
PedsQL – correlation between patients and parents

PedsQL correlation between patients and parents is shown in Figure 5.

In 76 cases (82%), the score assigned by the parent and the patient was in the same range; in 11 cases (11%), the parent's score was higher than the patient's; in 7 cases (7%), the parent’s score was lower than the patient's.

Correlation between meatal stenosis grading system and uroflowmetry is shown in Table 4.

• Grade 0: six patients (13%) had a pathological uroflowmetry and one (2.1%) had a borderline uroflowmetry.
• Grade 1: six patients (15%) had a pathological uroflowmetry and three (7.3%) had a borderline uroflowmetry.
• Grade 2: four patients (33%) had a pathological uroflowmetry and no patient had a borderline uroflowmetry.

**Table 4:** Correlation between meatal stenosis grading system and the results of performed uroflowmetry

Grade	Pathological urofluometry	Borderline urofluometry
0	6 (13%)	1 (2.1%)
1	6 (14.6%)	3 (7.3%)
2	4 (33.3%)	0

**Figure 6:**
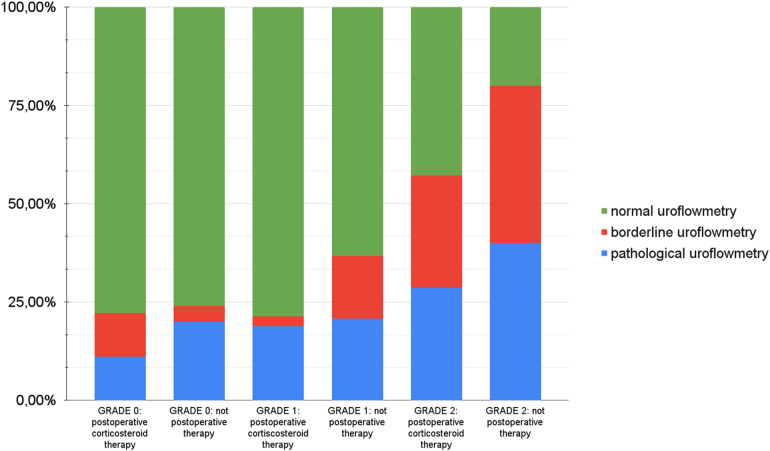
Correlation between postoperative corticosteroid topical therapy and the results of uroflowmetry based on meatal stenosis grade

Correlation between postoperative corticosteroid topical therapy and uroflowmetry based on meatal stenosis is shown in Figure 6.

• Grade 0: 19 patients received postoperative corticosteroid topical therapy. Of these, two patients (11%) had a pathological uroflowmetry and two (11%) had a borderline uroflowmetry. A total of 27 patients did not receive postoperative corticosteroid topical therapy; 5 of these patients (20%) had a pathological uroflowmetry and 1 (4%) had a borderline uroflowmetry.
• Grade 1: 16 patients received postoperative corticosteroid topical therapy. Of these, three patients (19%) had a pathological uroflowmetry and two (2.5%) had a borderline uroflowmetry. A total of 25 patients did not receive postoperative corticosteroid topical therapy; 5 of these patients (21%) had a pathological uroflowmetry and 4 (16%) had a borderline uroflowmetry.
• Grade 2: seven patients received postoperative corticosteroid topical therapy. Of these, two patients (29%) had a pathological uroflowmetry and two (29%) had a borderline uroflowmetry. Five patients did not receive postoperative corticosteroid topical therapy; two of these patients (40%) had a pathological uroflowmetry and two (40%) had a borderline uroflowmetry.

## Discussion

The incidence of LS has been increasing in the last few decades, not only in adults, but also in the paediatric population.^9,10^ Our results are in accordance with these data, with an increase in the number of circumcisions performed for phimosis with a histopathological diagnosis of LS from 2015 to 2021. This increase may represent a genuine increase in the incidence of the condition, because of an increase in infections due to changes in children's habits, or a biased increase due to a greater awareness of the condition among paediatric surgeons.^2^ Paediatric surgeons are nowadays more likely to complete a comprehensive foreskin examination, and, consequently, a larger group of foreskin samples are sent for histopathologic analysis.^11^

The most common treatment for LS is circumcision. Definitive surgery seems to be curative in most cases.^12^ In our study, all patients with phimosis and a clinical suspicion of LS underwent circumcision. In the literature, circumcision remains the gold standard treatment, despite the fact that there are no guidelines on the management of this disease in the paediatric population. An alternative surgical treatment option is the preputioplasty, a foreskin-preserving technique which has shown a success rate ranging from 50% to 81%.^13,14^ Vincent *et al* reported good outcomes with preputioplasty as a second-line treatment following failed topical corticosteroid therapy.^15^

The use of topical corticosteroid treatment remains a topic of debate. There is confusion in our practice about whether to use corticosteroids as pre or postoperative treatment. The literature also reports conflicting results.

Kiss *et al* and Vincent *et al* reported better aesthetic outcomes, but poor results if we consider the degree of foreskin inflammation due to LS after corticosteroid use alone.^15,16^ Bochove-Overgaauw *et al* recommended the systematic use of corticosteroids after circumcision in patients with a histopathological confirmation of LS.^12^ Based on our results, we report a relationship between no postoperative treatment with steroid and pathological or borderline pattern uroflowometry. Even if there is no statistically significant difference in either of three grades in term of urine flow, we would like to improve the use of postoperative steroids, especially in grade 2 cases, to reduce the incidence of meatal stenosis, which has been demonstrated to be the most important and common complication of LS. Meatal stenosis is defined as narrowing of the opening of the external urethral meatus to less than 2mm.^17^ Usually, it is asymptomatic in paediatric patients; however, serious consequences may be encountered, ranging from urinary obstruction to renal failure,^11^ and additional treatment such as serial urethral dilatation or surgical meatoplasty may be required.^11^ The incidence of meatal stenosis in our study is 4.7%, which is lower than that reported in the literature (7–19%).^10,18^

We had four cases of meatal stenosis that required urethral dilatation at follow-up. All of these patients had an improvement of urinary stream, confirmed by uroflowmetry, after a mean of 1.5 dilatations (1–3 dilatations). One patient underwent a home dilation programme with a resolution of meatal stenosis after three months. Despite the good results, we are aware that serial urethral dilatations under general anaesthesia have important implications for patients and their families, as well as a significant economic impact for the national health system.^2^ None of our patients underwent meatoplasty, contrary to the literature, which reports the use of meatoplasty as urinary reconstructive surgery associated with LS-linked meatal stenosis in 36% of paediatric patients.^11^ Based on our experience with asymptomatic meatal stenosis, we recommend serial assessment of patients diagnosed with LS, with clinical and uroflowmetry evaluation and long-term follow-up. We did not find a standardised postoperative management approach for these patients in the literature.^11^

## Conclusion

In conclusion, LS is a common condition in childhood. An increased incidence of the condition among the paediatric population over the last decade imposes the need for greater care in its diagnosis and management. There are currently no specific guidelines for a standardised management approach for LS in the paediatric population. Based on our experience and the results of this retrospective analysis, we can conclude that a correct diagnosis and appropriate medical and surgical treatment could improve long-term outcomes in these patients. We believe that topical corticosteroid treatment should be used for at least one month after surgery after the histopathological confirmation.

In terms of surgical treatment, circumcision remains, in our opinion, the gold standard to ensure the complete removal of LS. The use of topical corticosteroids as adjuvant therapy should be suggested, especially in case of grade 2 meatal stenosis, to reduce the incidence of complications.

## Limits of the study

The study was done in 2022. All patients were seen in 2022, but some were operated on in 2015 and some more recently in 2021. Therefore, the average time of follow-up is different and this needs to be taken into account.
